# Serum Extracellular Vesicle-Derived miRNAs in Patients with Non-Small Cell Lung Cancer—Search for Non-Invasive Diagnostic Biomarkers

**DOI:** 10.3390/diagnostics11030425

**Published:** 2021-03-03

**Authors:** Jolanta Kryczka, Monika Migdalska-Sęk, Jacek Kordiak, Justyna M. Kiszałkiewicz, Dorota Pastuszak-Lewandoska, Adam Antczak, Ewa Brzeziańska-Lasota

**Affiliations:** 1 Department of Biomedicine and Genetics, Medical University of Lodz, 92-213 Lodz, Poland; jolanta.kryczka@umed.lodz.pl (J.K.); justyna.kiszalkiewicz@umed.lodz.pl (J.M.K.); ewa.brzezianska@umed.lodz.pl (E.B.-L.); 2 Clinic of Thoracic Surgery, General and Oncological Surgery, University Clinical Hospital Named after the Military Medical Academy—Central Veterans’ Hospital, Medical University of Lodz, 90-549 Lodz, Poland; jacek.kordiak@umed.lodz.pl; 3 Department of Microbiology and Laboratory Medical Immunology, Medical University of Lodz, 92-213 Lodz, Poland; dorota.pastuszak-lewandoska@umed.lodz.pl; 4 Department of General and Oncological Pulmonology, Medical University of Lodz, 90-153 Lodz, Poland; adam.antczak@umed.lodz.pl

**Keywords:** non-small-cell lung cancer, biomarkers, miRNA, extracellular vesicles, progression

## Abstract

The aim of the study was a search for diagnostic and/or prognostic biomarkers in patients with non-small cell lung cancer (NSCLC) patients, based on circulating microRNAs (miRs: miR-23a, miR-361, miR-1228 and miR-let7i) in extracellular vesicles (EVs). Serum EVs were isolated from NSCLC patients (*n* = 31) and control subjects (*n* = 21). RNA was isolated from EVs and reverse transcription reaction was performed. Relative levels of miR-23a, miR-361, miR-1228 and miR-let7i were assessed in real-time qPCR using TaqMan probes. Analysis was based on the 2-ΔΔCT method. Statistically significant lower levels of miR-23a and miR-let7i were observed among NSCLC patients vs. control group: miR-23a, 0.054 vs. 0.107; miR-let7i, 0.193 vs. 0.369 (*p* = 0.003, *p* = 0.005, respectively). A receiver operating characteristic (ROC) curve analysis demonstrated the diagnostic potential of each individual serum EV-derived miRNA with an area under the curve AUC = 0.744 for miR-23a (*p* = 0.0003), 0.733 for miR-let7i (*p* = 0.0007). The decreased level of miR-23a in patients correlated with metastasis to lymph nodes and with AJCC tumor staging system. The results demonstrate that miR-23a and miR-let7i may prove clinically useful as significant, non-invasive markers in NSCLC diagnosis. Additionally, changing profile level of miR-23a that correlates with cancer development may be considered as an NSCLC progression marker.

## 1. Introduction

Lung cancer is the most common, aggressive malignancy, which is related to the highest number of deaths worldwide [1,2,3]. Lung cancer may be classified into two major groups based on histopathological diagnosis: Small-Cell Lung Cancer (SCLC—15%) and Non-Small-Cell Lung Cancer (NSCLC—85%) [3,4]. The NSCLC group is composed of Squamous Cell Carcinoma (SCC), Adenocarcinoma (ADC), and Large Cell Carcinoma (LCC) [1,2,3,4]. Due to no obvious symptoms suitable for early diagnosis and highly malignant potential, the 5-year survival rate of NSCLC remains extremely low at 15–20%, with the average survival time of 8–12 months [1,5].

Experimental and clinical studies showed that qualitative composition of extracellular vesicles (EVs) may serve as reliable biomarkers and early diagnostic factors of cancer disease [6].The EVs are composed of: exosomes, microvesicles and apoptotic bodies [7]. Microvesicles, 150–1000 nm in size, originate from invagination of the plasma membrane capturing cytoplasmic contents. Exosomes are smaller in size, ranging from 40 to 150 nm, and derive from early endosomes. Apoptotic bodies measure 100–5000 nm in diameter, and are vesicles that originate from dying cells as they disintegrate [7]. EVs collectively are secreted by a heterogeneous group of cells in physiological and pathological conditions, including tumor cells [8,9,10]. As functional carriers for intercellular communication, they contain thousands of active particles, such as various types of lipids, proteins, mRNA and microRNAs (miRNAs) [8,9]. Furthermore, they exert good stability in most body fluids, thus enabling delivery to the target tissue [6]. EVs are mediators of the constant communication between tumor cells and surrounding stromal cells during tumor invasion. They are recognized as a bioactive reservoir of oncogenic proteins such as tyrosine kinase. For example, in tumorgenesis of colon cancer, they trigger the phenotypic conversion of progenitor smooth muscle cells into matrix metalloproteinases 1 (MMP1) secreting tumor-promoting cells [11].

MicroRNAs transported in EVs are highly conserved, non-coding, single-stranded, endogenous RNA molecules (21–25 nucleotides), that are transcribed by RNA polymerase II into pri-pre-miRNA [1,5,12]. They can bind to the 3′-untranslated regions (3′UTR) of the target gene and regulate its expression at the post-transcriptional level [13].

Numerous data show that miRNAs may act as oncogenes or tumor suppressors playing an important role in the regulation of the expression of genes involved in the carcinogenesis, metastasis and invasion, thus causing lung cancer reemission [4,12,14]. Hence, such miRNAs are referred to as “oncomiRs” [15]. Aberrant expression of miRNAs is observed in different materials isolated from patients with NSCLC, such as plasma, serum and lung tissue and also from patient-derived cell lines [4,16,17,18,19,20,21,22]. Furthermore, no potential biomarkers have aroused as much interest as miRNAs, which are considered as a future early diagnostic and prognostic tool [22,23,24].

The expression of miRNAs: miR-23a, miR-361, miR-1228 and miR-let7i has been extensively studied in many types of human cancer (including lung cancer) and the significant dysregulation of their expression levels was observed in different biological materials isolated from patient [4,5,15,23,25,26,27,28]. However, the potential prognostic/diagnostic EV-derived miRNA panel has not been assessed.

There are several studies on the role of miR-23 as an oncogene in various types of cancers, mainly due to its stimulatory effect on cell migration and invasion [23,29]. MicroRNA profiling analysis points that miR-23a cluster is significantly upregulated in breast cancer, hepatocellular carcinoma (HCC), pancreatic adenocarcinoma and lung cancer [16]. MiR-23a plays a vital role in the regulation of TGF-β-induced epithelial to mesenchymal transition (EMT) by targeting E-cadherin, as shown in lung cancer cells [29]. Additionally, upregulated expression level of miR-23a in NSCLC cell lines and tissue samples leads to increased drug-resistance, which further proves its prognostic usefulness [22,23].

The research conducted on the lung cancer cell lines showed that miR-1228 acts as a regulator of the tumorgenesis progress and cell response to environmental stress [25]. Significant downregulation of miR-1228 was observed in gastric cancer tissues compared with normal tissues [27]. Additionally, miR-1228 is extensively involved in metabolism-related signal pathways, as well as in organ morphology regulation, implying that miR-1228 could function as a housekeeping gene [30].

Down-regulated expression of miR-361, which acts as tumor suppressor, is observed in gastric cancer tissues and cancer cell line models, colorectal cancer, prostate cancer, cutaneous squamous cell carcinoma and lung cancer [5,25,31]. In NSCLC tissue, the expression level of miR-361 is significantly lower as compared to adjacent tissues thus suggesting its potential inhibitory role in cancer progression [32]. Kaplan–Meier analysis of NSCLC patient survival, based on miR-361 expression in cancer tissue, proved that its low expression significantly decreases patients survival time, serving as a predictor factor of clinical outcome [33].

The let7 family is known to be a tumor suppressor, with significantly down-regulated expression observed in several cancers [15,34]. As found in NSCLC tissue and NSCLC cell line models, it is involved in the regulation of key metastatic processes such as: proliferation, invasion and apoptosis [35] via down-regulation of a number of oncogenes (*KRAS*, *c-MYC*, *CDK6*, *HOXA9*, *TGFBR1*, *BCL-XL*, and *MAP4K3*) and key cell cycle regulators (CDK6, CDC25A, and cyclin-D2) [15,34,35,36,37]. The early let-7 down-regulation in rat cancer tissue has been recognized as a fundamental step in cigarette smoke-induced lung carcinogenesis [38]. Additionally, it has been shown that lung cancer patients with low let-7 expression in cancer tissue had significantly shorter survival time compared to its moderate or high expression [39].

In the light of many reports focused on evaluation of the circulating miRNA expression in patients with NSCLC, it seems reasonable to conduct further research in this area in order to improve therapy and diagnosis of NSCLC.

The aim of the research is to specify the level of selected miRNAs in EVs: miR-23a, miR-361, miR-1228 and miR-let7i among NSCLC patients compared with health controls in order to search for NSCLC biomarkers, useful for diagnosis and monitoring of the disease.

## 2. Materials and Methods

### 2.1. Research Ethics

This study was conducted in accordance with Good Clinical Practice and the principles of the Helsinki Declaration. The protocols of this study were approved by the Bioethics Committee of the Medical University of Lodz (resolution No. RNN/85/18/KE of 13 March 2018). All participants signed an individual consent form for participation in the study.

### 2.2. Patient and Control Group Selection

A total of thirty-one (*n* = 31) patients with a confirmed diagnosis of NSCLC, 20 men and 11 women (mean age, 67 ± 7.31 years, range 51–79 years) were enrolled into the study. Patients were qualified in the Department of Thoracic Surgery, General and Oncologic Surgery, Military Medical Academy Memorial Teaching Hospital of Medical University of Lodz—Central Veterans’ Hospital, Lodz, Poland, between 2018 and 2019. The smoking status data was collected. A total of 14 patients were ex-smokers, 12 current smokers and 5 patients were never smokers. For further correlation analyses, smokers were divided into two groups: ≤40 PYs (*n* = 10) and >40 PYs (*n* = 13), depending on the numbers of pack-years (PY, 1 Pack Year = 20 cigarettes smoked per day for 1 year; according to NCI Dictionary of Cancer Terms) [40]. The pack-year smoking history was not available for 3 patients. The tissue samples were subjected to postoperative histopathological evaluation and classified according to the AJCC and TNM staging system (pTNM) [41]. The results of the histopathological verification of lung cancer, based on pathomorphological reports, are summarized in Table 1.

The control group (*n* = 21) consisted of age-matched (mean age, 65 ± 11.52 years, range 42–86 years) healthy female volunteers who participated in the screening tests at the Outpatient Clinic of Osteoporosis; Regional Center of Menopause and Osteoporosis, Military Medical Academy Memorial Teaching Hospital of Medical University of Lodz—Central Veterans’ Hospital, Lodz, Poland. The exclusion criteria included osteoporosis, diagnosis of cancer and the presence of other comorbidities that could significantly affect the study results.

Regarding age, there were no statistically differences between NSCLC patients and control group (*p* = 0.8389; Mann–Whitney U-test).

### 2.3. Serum Collection

A total of four milliliters of venous blood was collected into tubes without anticoagulant and left at room temperature until clot formation (about 30–60 min). Next, the samples were centrifuged (1200× *g*, 10 min, 23 °C), and serum was separated into new sterile tubes, frozen, and stored at −20 °C. Serum from all (*n* = 31) NSCLC patients was collected before the surgery. Serum from 21 patients (volunteers) was obtained as a control group, during a routine medical visit.

### 2.4. Extracellular Vesicles Isolation from Serum

EVs isolation from frozen human serum was performed using a Total Exosome Isolation (from serum) kit (Life Technologies, Carlsbad, CA, USA), according to the manufacturer’s protocol. Serum samples were thawed by placing them in a 25 °C water bath. To remove the cells and residues from the serum, samples were centrifuged at 2000× *g* for 30 min. Then, 500 μL of the supernatant containing the clear serum was moved to a new tube and 0.2 volumes of the Total Exosome Isolation reagent was added. The serum/reagent mixture was mixed well by vortex until there was a homogenous solution. The samples were incubated at 2–8 °C for 30 min, and afterwards they were centrifuged at 10,000× *g* for 10 min at room temperature. The supernatant was removed and the pellet containing the EVs was suspended in 200 μL of 1× PBS and stored at 2–8 °C for a week or frozen at −20 °C until RNA isolation.

### 2.5. RNA Isolation from EVs, Qualitative and Quantitative RNA Assessment

RNA isolation was performed using a Total Exosome RNA & Protein Isolation Kit (Life Technologies, Carlsbad, CA, USA), according to the manufacturer’s protocol.

Qualitative and quantitative evaluation of the isolated RNA was performed by spectrophotometric method, measuring the absorbance with the Eppendorf BioPhotometerTM Plus (Eppendorf, Hamburg, Germany), at 260/280 nm wavelengths. RNA with a 260/280 nm ratio in a range of 1.8–2.0 was considered to be of high quality and used to synthesize cDNA. Then RNA was aliquoted and frozen at −80 °C until the real-time polymerase chain reaction (qPCR) was performed.

### 2.6. Reverse Transcription, Real-Time Quantitative PCR and miRNAs Level

cDNA was transcribed from 5 μL (10 ng) of template RNA, using TaqMan^®^ MicroRNA Reverse Transcription Kit (Applied Biosystems, Carlsbad, CA, USA) and 3 μL of specific RT primers (small RNA-specific RT primers) included in individual TaqMan^®^ MicroRNA Assays: hsa-miR-23a (AUCACAUUGCCAGGGAUUUCC, ID 000399), hsa-miR-361 (UUAUCAGAAUCUCCAGGGGUAC, ID 000554), hsa-miR-1228 (UCACACCUGCCUCGCCCCCC, ID 002919) and hsa-let7i (UGAGGUAGUAGUUUGUGCUGUU, ID 002221) (Applied Biosystems, Carlsbad, CA, USA). Reverse Transcription (RT) master mix contained: 0.15 μL 25× dNTP Mix (100 mM), 1 μL MultiScribe™ Reverse Transcriptase (50 U/μL), 1.5 μL 10× RT buffer, 0.19 μL RNase Inhibitor (20 U/μL), and 4.16 μL nuclease-free water. Negative control was included in each RT reaction, containing no RNA (No RT control). RT reaction, in a total volume of 15 μL, was performed in a Personal Thermocycler (Eppendorf, Germany). The RT reaction conditions were as follows: 30 min at 16 °C, 30 min at 42 °C, then 5 min at 85 °C and 4 °C hold. RT products were stored at −20 °C until further analysis.

The levels of the studied miRNAs were assessed in real-time quantitative PCRs (qPCR) using TaqMan^®^ probes (Applied Biosystems, Carlsbad, CA, USA). A total reaction mixture volume of 20 μL contained: 1.33 μL RT Product, 1 μL TaqMan^®^ MicroRNA Assays, 10 μL KAPA PROBE FAST qPCR Master Mix (2X) ABI Prism™ (Kapa Biosystems Ltd., London, UK), and 7.67 μL nuclease-free water (Applied Biosystems, Carlsbad, CA, USA). The qPCR of miRNAs was performed on 96-well plates and assessed in the Applied Biosystems 7900 HT Fast Real-Time PCR System (Applied Biosystems, Carlsbad, CA, USA) for 39 cycles, with an annealing temperature of 60 °C; this was repeated three times for each sample.

MiRNAs level analysis was performed using DataAssist v3.01 (Life Technologies, Carlsbad, CA, USA), based on the global normalization method [43]. The software calculates the mean level of all the miRNAs. The median CT of those assays is used as the normalizer to perform the ΔCt per a sample. Next, the formula ΔΔCt = ΔCT test sample—ΔCT calibrator sample is calculated, where RNA isolated from a healthy patient (reference patient) serves as a calibrator, for which the RQ (relative quantification) value is considered equal to 1.

### 2.7. Statistical Analysis

The statistical analysis was carried out using the Statistica 13.1 program (StatSoft, Cracow, Poland). The Shapiro–Wilk test showed that data were not normally distributed. In order to look for the statistical significance between the analyzed groups, Mann–Whitney U-test and/or Kruskal–Wallis tests were used, depending on the size of the groups. Neuman–Keuls’ multiple comparison test was used to identify possible significant differences in RQ values between the individual variables. The area under the receiver operating characteristic (ROC) curve was assessed to test the sensitivity and specificity of the studied miRNAs. The area under the curve (AUC) was resolved with a 95% confidence interval (CI). For all statistical analysis, the level of statistical significance was assumed at *p* < 0.05. The RQ values for the studied miRNAs are presented as mean ± standard deviation (median).

## 3. Results

### 3.1. The Levels of NSCLC EV-Derived miRNAs vs. Control

The qPCR results (RQ values) for miRNAs were obtained for all NSCLC patients and control samples. For details, see Supplementary Tables S1 and S2. In comparison to calibrator, miR-let7i, miR-23a and miR-361 revealed RQ < 1.0 in the majority of samples: 98–100%, depending on the miRNA. Among study samples, lower RQ values (RQ < 0.5 indicating < two fold lower level of miRNA) was observed in 86.5% NSCLC samples for miR-let7i, in 96.2% samples for miR-361 and in 100% samples for miR-23a. The level of miR-1228 was higher (RQ > 1) in all (100%) of study samples compared to the calibrator. Moreover, 96.2% of samples showed RQ > 2.0, thus indicating more than a two-fold higher level of this miRNA in NSCLC samples compared to the calibrator. The results representing RQ values for all miRNAs and the number of samples with lower/higher miRNA levels in NSCLC vs. calibrator are presented in Table 2.

**Figure 1:**
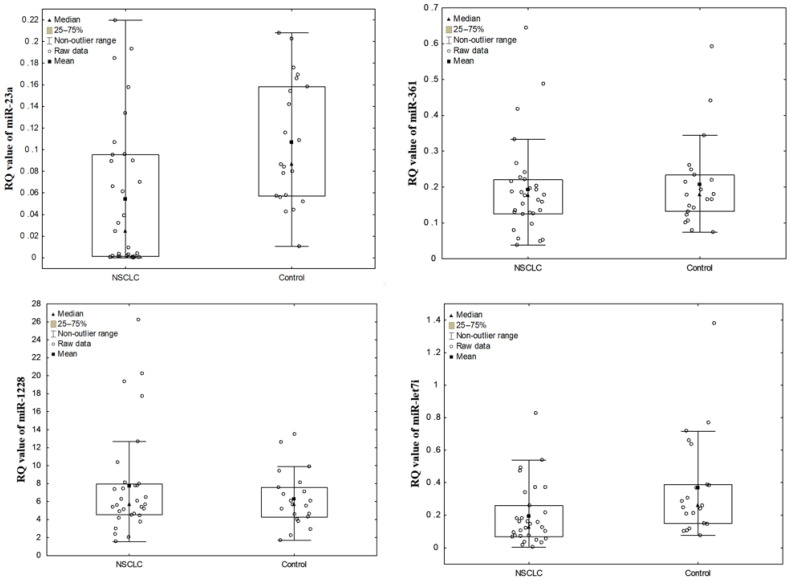
The dot plots, representing miRNA levels (RQ values) in NSCLC samples and control group.

The relative levels of the studied miRNAs were compared between NSCLC patients and controls. MiR-23a and miR-let7i were significantly down-regulated in NSCLC patients in comparison to control group (*p* = 0.0030 and *p* = 0.0047, respectively, Mann-Whitney U-test) (see Figure 1). Statistical analysis did not reveal significant differences in the relative levels of miR-361 and miR-1228 between NSCLC and control samples (*p* = 0.6278 and *p* = 0.6278, respectively, Mann–Whitney U-test).

Receiver operating characteristic (ROC) curve analyses were performed to calculate the diagnostic value of the studied miRNAs in NSCLC patients compared to healthy control group. A measure of the overall performance of a diagnostic test is the area under the ROC curve (AUC), which was calculated to estimate the specificity and sensitivity of miRNA to diagnose patients with NSCLC. The choice of an optimal cutoff point for dis-criminating between the NSCLC patients and the healthy controls was determined by the Youden index (J). J is the maximum vertical distance between the ROC curve and the diagonal reference line and is calculated as J = maximum (sensitivity + specificity − 1) [44].

**Figure 2:**
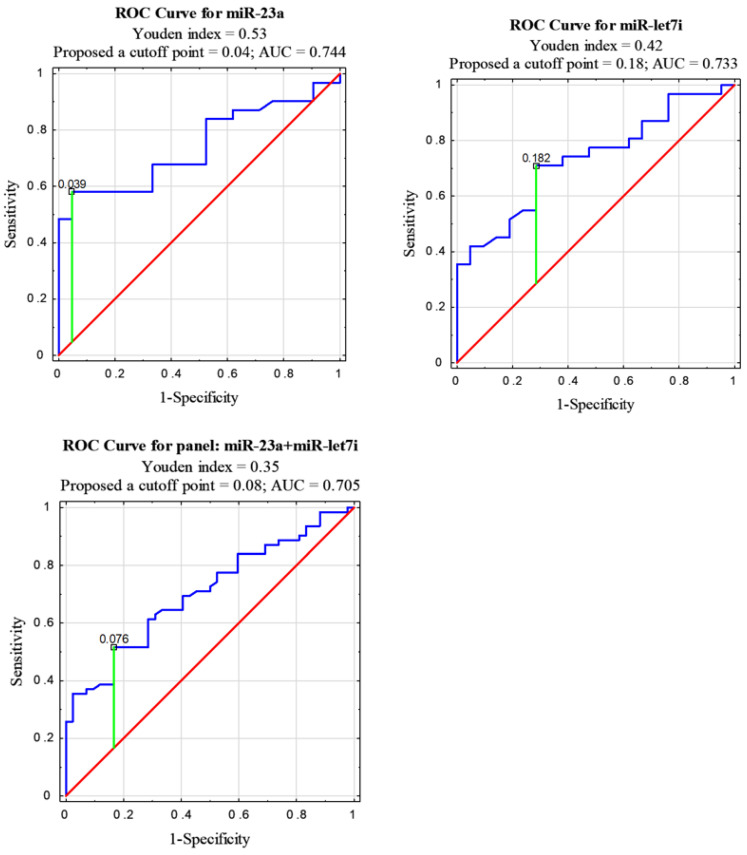
Receiver operating characteristic (ROC) curve analysis for serum EVs miRNA diagnostic potential in differentiating NSCLC and control.

The levels of miR-23a and miR-let7i in the NSCLC samples were significantly lower than in the healthy controls. These two miRNAs can differentiate NSCLC patients from controls, with AUC = 0.744 for miR-23a and 0.733 for miR-let7i. An optimal cutoff point was indicated at 0.039 with a sensitivity of 58% and a specificity of 95% (*p* = 0.0003) for miR-23a and 0.182 with a sensitivity of 71% and a specificity of 71% (*p* = 0.0007) for miR-let7i. AUC for miR-361 classifier was 0.538 and an optimal cutoff point was indicated at 0.57 with a sensitivity of 13% and a specificity of 0% (*p* > 0.05). AUC for miR-1228 classifier was 0.459 and an optimal cutoff point was indicated at 8.089 with a sensitivity of 81% and a specificity of 24% (*p* > 0.05) (see Figure 2 and Table 3).

All tested miRNAs were also analyzed as combined (in a panel). The highest AUC = 0.705 among the proposed panels was obtained for miR23a + miR-let7i. An optimal cutoff point for this panel was indicated at 0.076 with a sensitivity of 52% and a specificity of 83% (*p* = 0.00001) (see Figure 2). Combined miR-23a and/or miR-let7i with either miR-361 and/or miR-1228 gave an AUC < 0.6 (see Table 3).

### 3.2. Correlations between EV-Derived miRNA Levels and Clinicopathological Characteristics of NSCLC Patients

The levels of miRNA were evaluated in correlation with such clinicopathological data as: age at time of diagnosis, sex and smoking history as well as histopathological characteristics of tumors (according to pTNM and AJCC classifications and NSCLC subtypes). For details, see Supplementary Table S3.

**Figure 3:**
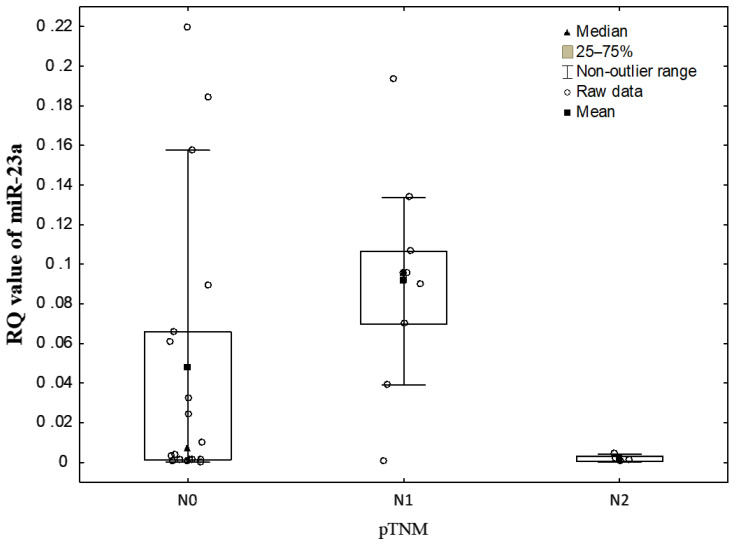
The dot plots representing miR-23a levels (RQ values) in the studied NSCLC groups classified according to the degree of lymph node involvement in pTNM classification.

The statistically significant differences in the RQ values of miR-23a, depending on the degree of lymph node involvement (N0 vs. N1 vs. N2 according to pTNM staging) were noted (*p* = 0.0313, Kruskal-Wallis test). Significantly lower miR-23a level was found in N2 than in N1 (*p* = 0.0277, Neuman–Keuls’ multiple comparison test) (see Figure 3).

According to the AJCC classification the relationship between the RQ values of miR-23a and the stage of tumor was revealed (*p* = 0.0391, Kruskal-Wallis test). The Neuman–Keuls’ multiple comparison test found a borderline statistically significant decrease in miR-23a level in the stage III when compared to the stage II (*p* = 0.0696).

Any statistically significant correlations were found between the RQ values of the studied miRNAs and the clinical features of NSCLC patients, i.e., patient age, sex, or history of smoking assessed as PYs (*p* > 0.05; Kruskal-Wallis test). Similarly, no associations were found with the histopathological NSCLC subtypes and tumor size (pT) according to the pTNM staging (*p* > 0.05; Mann–Whitney U-test, Kruskal–Wallis test).

## 4. Discussion

NSCLC is the most common cause of cancer deaths worldwide, mainly due to the lack of effective early detection methods. This leads to late diagnosis and poor prognosis resulting in the inability to treat the advanced metastatic disease [45,46]. Currently, clinical factors such as age, tumor stage, smoking history, histological type of cancer and treatment modality are conventionally used as predictors for the prognosis of NSCLC [47]. There is a real necessity to search for reliable and non-invasive diagnostic and/or prognostic markers of lung cancer.

Liquid biopsy is a marginally invasive method that can detect circulating tumor cells and tumor-derived nucleic acids (e.g., cell-free DNA and miRNAs) in the blood of patients with lung cancer [48]. However, an increasing interest in potential clinical application of EVs has recently emerged. They may be recommended as ideal biomarkers, because of their considerable stability in blood as well as their protective role in relation to the content of the informative biological material (including mRNAs, miRNAs, and proteins) derived from the cancer cells. The growing number of studies focused on the biological function and implications of EVs load during cancer progression and metastasis has been published [47].

In our research, due to the limited amount of material, we isolated EVs from human serum. The method is based on the polymer precipitation technique. The polymers bind water molecules, allowing the reagent to remove less soluble reagents from the solution and isolate them in the sediment by brief centrifugation. EVs after spin remain in pellets resuspended in PBS. The purity of the sample allows for further RNA isolation. The widely used approach isolation of exosomes from body fluids is based on ultracentrifugation in combination with sucrose density gradients or sucrose cushions to float the relatively low-density exosomes away from other vesicles and particles. These protocols can range in time from 8 to 30 h and a require large amount of material for successful isolation of exosomes [49]. However, comprehensive study of Helwa I. et al. supported the use of the commercial kits including Invitrogen Total Exosome Isolation Reagent as an adequate alternative to ultracentrifugation, even with limited amounts of heterogeneous biological starting material [50]. Due to limited amount of material no further characterization of the isolated vesicles was performed.

Tumor cells secreting large amounts of EVs (significantly more than normal cells of the same origin) seem to be essential from diagnostic and a prognostic point of view. In patients with diagnosed cancer, an isolated EVs fraction from blood is mainly composed of tumor derived particles [48]. Therefore, miRNAs derived from cancer EVs play a significant role in the transduction of various signals between tumor cells. They are recognized as a regulator of distant metastatic niche and drug resistance [47,48].

Based on the above reports, we analyzed the levels of three miRNAs, miR-26a, miR-29b and miR-519d in NSCLC tissue samples, and evaluated their diagnostic potential in NSCLC carcinogenesis [46]. As a continuation of this research, we have selected other miRNAs that could enlarge the possible panel of NSCLC diagnostic biomarkers.

In the present study, we determined the profile of levels of four miRNAs (miR-let7i, miR-23a, miR-361, miR-1228), incorporated into blood circulating EVs and evaluated their potential role as diagnostic and/or prognostic biomarkers of NSCLC. We observed decreased levels of three out of four studied miRNAs (i.e., miR-let7i, miR-23a and miR-361) and an increased level of one miRNA (miR-1228) in NSCLC patients compared to reference patient. Significant differences were found for miR-23a and miR-let7i compared control group.

The decreased level of miR-23a revealed in our study is in contrary to the results of other authors, who found its higher expression in plasma and different NSCLC cell lines [20,21,51]. However, Wei-Qing Qu et al. showed that in lung tissues samples obtained from NSCLC patients, the percentage of probes with a reduced miRNA-23a expression level was similar to that with an up-regulated miR-23a expression level [4]. Moreover, in our study, miRNA-23 was isolated from EVs, while in the above-mentioned studies, other biological materials were used, such as blood, plasma, lung tissue or cell lines, which may affect the differences in the obtained results. The selective packaging of biological active molecules, including miRNAs, into EVs may drastically change during tumorgenesis [47]. Interesting and important results were obtained by Jin X. et al., who demonstrated that the expression level of the isolated miRNAs from plasma exosomes differed significantly when compared to the miRNA isolated from exosome-depleted plasma. Much lower levels of miRNAs were observed in EVs. However, total circulating plasma miRNAs may be composed of endogenous cellular miRNAs derived from debris from all cell types [18].

Interestingly, Kaplan–Meier meta-analysis of miR-23a expression level in various tissue cancers, including lung cancer, proved that its up-regulated level may be considered as a prognostic marker [4,23]. Several studies on lung cancer cell lines showed that particularly miR-23 may serve as an excellent marker for poor prognosis of lung cancer [23,29] through its stimulatory role in migration and invasion of cancer cells [52]. MiR-23a via targeting E-cadherin may regulate TGF-β-induced EMT [20]. A study conducted by others confirmed a significant difference in the expression levels of plasma miRNA-23a between the different stages of the disease. The plasma miRNA-23a expression levels in the lung cancer patients with distant metastasis were significantly higher than those in the patients without metastasis, and the expression of miRNA-23a was significantly associated with tumor size, but not significantly related to lymph node metastasis [33]. On the contrary, we observed a statistically significant lower level of miR-23a in N2 group compared to N1. However, our test groups were small and this result requires further confirmation on the larger groups of patients with diagnosed N1 and N2 stage (according to TNM staging system). Furthermore, in our research most patients with diagnosed NSCLC were in early stage cancer disease according TNM classification (70% patients were in I and II stage), while in the study of Hetta et al. 84.4% of patients were in advanced cancer stage (III, IV stage) [20].

An increasing number of publications confirms also the significance of miR-let7i and its correlation with the staging, differentiation, and metastasis of cancer [53]. Therefore, in our study, we also analyzed miR-let7i level and found it decreased in NSCLC patients compared to control group. Our results are consistent with the data obtained by others, who focused on NSCLC patients or lung cancer cell lines [54,55]. According to published data, the expression level of miR-let7i is reduced in various lung cancer cell lines and pulmonary tumors, compared to normal lung samples [50,51,52]. It is consistent with the recognized role of let7 family as tumor suppressor [15,55,56,57]. During carcinogenesis, the chromosomal region involving miR-let7 *locus* is often deleted, resulting in the reduced expression of this miRNA, and thus associated with poor prognosis [37]. The putative mechanisms of let7 down-regulation in cancer include not only genetic alterations, but also regulation of *RAS* and *MYC* oncogenes, direct targeting of *DICER* mRNA and cell proliferation control in a cyclin-dependent manner [58]. It was recognized that its cancer tissue level may be considered as a survival prognostic marker for surgically treated lung cancer patients [55]. Furthermore, miR-let7i is mainly involved via down-regulation of a number of oncogenes (*RAS*, *c-MYC*, *CDK6*, *HOXA9*, *TGFBR1*, *BCL-XL*, and *MAP4K3*) in the negative regulation of metastatic processes [35]. MiR-let7i acts as a negative regulator of the *RAS* family and its level in cancer tissue is reversely correlated with cancer progression [15,35,36,37,55]. Surprisingly, in our study, we did not notice any significant correlation between miR-let7i level and the cancer staging system (according to the pTNM and AJCC classifications). However, the limitation of our research that may contribute to the lack of statistical significance of the results was the small number of groups which we compared. Similarly to our research, there are about 16% of other studies which have the limited statistical power [59].

According to other authors, the modified miR-let7 expression was found in up to 50% of cells in lung adenocarcinoma cell lines and tissue samples, which led to increased cancer progression [54,55]. It was confirmed that the overexpression of miR-let7 may repress proliferation of A549 lung adenocarcinoma cells, whereas administration of antisense molecules targeting let7 increased their proliferation rates [54,55]. In turn, we observed the down-regulated miR-let7 level in all study histopathological NSCLC subtypes (SCC, ADC and LCC) with the highest level in ADC samples. In our study, there were no correlations between histopathological NSCLC subtypes and the changed level of miR-let7 and miR-23. Similarly, data published by others did not confirm the significant difference in the serum EV-derived miRNA-23a levels in the lung cancer patients with different histopathological types [20].

Using the ROC curve analysis for miR-23a and miR-let7i separately, we found miR-23a to be more specific and miR-let7i more sensitive. The combination of miR-23a and miR-let7i shows greater specificity than miRNAs analyzed individually and similarly, moderate diagnostic value for NSCLC. Thus, these two miRNAs may have potential for further evaluation as biomarkers in the early diagnosis of NSCLC.

In our study we evaluated EV-derived miRNAs levels, considering such factors as age at time of diagnosis, sex and smoking history. We did not notice any significant differences in the level of miR-23a and miR-let7i in relation to those parameters.

Cigarette smoking is a significant risk factor for lung cancer. High expression of miR-23a in NSCLC tissues was shown to be significantly associated not only with the advanced stage of diseases but also independently with the smoking status. Current data suggests that smoking influences different molecular alterations, including dysregulation of miRNA expression level in tumor tissue, body fluids and serum in lung cancer [60,61]. In our study, 84% of patients were ex-smokers or current smokers while levels of miR-23a were decreased, without statistically significant differences in both these groups. Our results do not confirm the research of others who documented a positive correlation between miR-23a up-regulated expression and smoking [61,62]. Interestingly, recent reports prove that the level of serum let7 is down-regulated in smoking patients, with a significant up-regulation of cyclin F that is directly targeted by let7 [28,63]. Our results are similar to those of other authors. We confirmed the down-regulation of miR-let7i in all patients with diagnosed NSCLC, including smokers but without statistical significance, also regarding ex-smokers or current smokers. The correlation between smoking status and miRNAs level is difficult to establish, especially considering the fact that EVs are subjects of selective content packaging of different biological signaling molecules including miRNAs [47].

Our study was limited by: small number of miRNAs assessed, small number of patients and no extra-cellular vesicles characterization.

## 5. Conclusions

EVs as potential source of biomarkers are currently strongly considered, due to their easy accessibility and high stability. They contain information derived from the original tissues and as such, reflects this information using different molecules, including miRNA. Establishing of qualitative and quantitative molecular signatures of specific EVs is necessary in order to apply EV-based tumor diagnostic and cancer development monitoring.

We confirmed the association between modified levels of EV-derived miRNAs and NSCLC as compared to healthy controls. Based on our data, we conclude that miR-23a and miR-let7i may be considered as potential non-invasive biomarkers differentiating healthy people from patients with diagnosed NSCLC. Nevertheless, this data requires further confirmation on a larger group of patients that would provide higher statistical significance.

## Figures and Tables

**Table 1:** Histopathological features of NSCLC samples.

Features	*n* (%)
Histopathological type	*n* = 31
SCC	15 (48.5)
ADC	15 (48.5)
LCC	1 (3)
Tumor size; T (pTNM staging system)	*n* = 31
T1a + T1b + T1c	10 (32)
T2a + T2b	12 (39)
T3 + T4	8 (26)
Tx	1 (3)
Intrathoracic lymph node involvement; N (pTNM staging system)	*n* = 31
N0	18 (58)
N1	9 (29)
N2	4 (13)
Malignant stage (AJCC staging system)	*n* = 30 *
Stage I	12 (40)
Stage II	9 (30)
Stage III	9 (30)

* AJCC was not determined in one patient due to the lack of T (Tx) feature. SCC—squamous cell carcinoma; ADC—adenocarcinoma, AJCC—American Joint Committee on Cancer Staging according to the IASCLC Staging Project 8th ed. (2017) [42] Cancer; pTNM—post-operative Tumor Node Metastasis staging system according to the WHO Histological Typing of Lung Tumor.

**Table 2:** RQ values for all studied miRNAs in serum EVs of patients with NSCLC and the control group.

miRNA	Sample	RQ Value Mean ± SD (Median)	Range of RQ Value	Number (%) of Samples with	
				RQ Value > 1	RQ Value < 1
miR-23a	NSCLC	0.054455 ± 0.066 (0.024)	0.000100–0.219600	0 (0)	31 (100)
	Control	0.107062 ± 0.058 (0.087)	0.010600–0.208100	0 (0)	21 (100)
miR-361	NSCLC	0.193529 ± 0.130 (0.177)	0.038100–0.644600	0 (0)	31 (100)
	Control	0.206724 ± 0.124 (0.177)	0.074100–0.592100	0 (0)	21 (100)
miR-1228	NSCLC	7.725716 ± 5.756 (5.661)	1.551500–26.23720	31 (100)	0 (0)
	Control	6.264948 ± 3.098 (5.637)	1.689000–13.48830	21 (100)	0 (0)
miR-let7i	NSCLC	0.192923 ± 0.187 (0.128)	0.004200–0.826300	0 (0)	31 (100)
	Control	0.369348 ± 0.312 (0.260)	0.077400–1.378300	1 (95)	20 (95)

**Table 3:** Diagnostic values with area under the curve (AUC) data from ROC curves and 95% confidence intervals (CI) for serum EV-derived miRNAs (individually and in panels).

miRNA	AUC	95% CI	*p* Value
miR-23a	0.744	0.611–0.877	0.0003
let7i	0.733	0.598–0.867	0.0007
let7i + miR-23a	0.705	0.606–0.803	0.00001
let7i + miR-361	0.651	0.546–0.756	0.0048
let7i + miR-23a + miR-361	0.650	0.565–0.735	0.0005
miR-23a + miR-361	0.612	0.505–0.718	0.0406
let7i + miR-23a + miR-361 + miR-1228	0.582	0.505–0.659	0.0357
miR-23a + miR-1228	0.551	0.441–0.662	0.364
let7i + miR-1228	0.548	0.438–0.658	0.392
miR-361	0.538	0.378–0.698	0.6379
miR-361 + miR-1228	0.499	0.387–0.612	0.992
miR-1228	0.459	0.3–0.619	0.619

## Data Availability

The research date well be available upon request.

## Supplementary Materials

The following are available online at https://www.mdpi.com/2075-4418/11/3/425/s1, Table S1: The levels of miRNAs (RQ values) in NSCLC samples group, Table S2: The levels of miRNAs (RQ values) in control group, Table S3: Comparison of miRNAs levels and clinicopathological data as: age at time of diagnosis, sex and smoking history as well as histopathological characteristics of tumors (according to pTNM and AJCC classifications and NSCLC subtypes); Data are is presented as mean ± standard deviation (median).
