# Supplementary material for: Serum Extracellular Vesicle-Derived miRNAs in Patients with Non-Small Cell Lung Cancer—Search for Non-Invasive Diagnostic Biomarkers
Source: Diagnostics (Basel). 2021 Mar 3;11(3):425. doi: 10.3390/diagnostics11030425 (PMC7998231; doi:10.3390/diagnostics11030425)
Supplement: Supplementary file 1 [file diagnostics-11-00425-s001.pdf]

Table S1. The levels of miRNAs (RQ values) in NSCLC samples group.

Sample No.	miR-let7i	miR-1228	miR-23a	miR-361
1	0.1622	6.2868	0.0957	0.1591
2	0.3414	6.0746	0.1842	0.1646
3	0.2141	4.4213	0.1575	0.2262
4	0.1276	17.7062	0.0244	0.0565
5	0.8263	3.7514	0.2196	0.2666
6	0.0661	20.2151	0.001	0.0495
7	0.4725	6.5096	0.0895	0.1536
8	0.1455	8.089	0.0659	0.1236
9	0.1815	7.441	0.0898	0.1344
10	0.0743	7.7622	0.0323	0.1288
11	0.3716	2.9993	0.1933	0.3334
12	0.2593	10.367	0.061	0.0965
13	0.1227	19.3806	0.0006	0.0516
14	0.4919	26.2372	0.0042	0.0381
15	0.0559	5.175	0.0698	0.1932
16	0.5393	4.5263	0.1066	0.2209
17	0.108	5.0975	0.039	0.1962
18	0.1559	4.9007	0.0954	0.2041
19	0.3713	5.3627	0.1338	0.1865
20	0.0764	5.3746	0.0036	0.1861
21	0.0717	5.6611	0.0014	0.1766
22	0.1594	4.644	0.003	0.2153
23	0.0476	12.6911	0.0005	0.0788
24	0.0179	2.0493	0.0004	0.488
25	0.0042	7.743	0.0001	0.1291
26	0.1019	7.9513	0.001	0.1258
27	0.0377	4.1524	0.0012	0.2408
28	0.0327	7.3671	0.0015	0.1357
29	0.0681	1.5515	0.0003	0.6446
30	0.0944	2.3986	0.0096	0.4169
31	0.1812	5.6097	0.0019	0.1783

Table S2. The levels of miRNAs (RQ values) in control group.

Sample No.	miR-let7i	miR-1228	miR-23a	miR-361
1	0.7161	1.689	0.1756	0.5921
2	0.3038	6.0587	0.0798	0.1651
3	0.2134	8.1024	0.0572	0.1234
4	0.366	4.6669	0.1085	0.2143
5	0.108	7.0626	0.0106	0.1416
6	1.3783	2.2715	0.2024	0.4402
7	0.6365	5.5276	0.0866	0.1809
8	0.6591	2.904	0.1691	0.3444
9	0.1463	13.4883	0.0441	0.0741
10	0.7689	3.8362	0.166	0.2607
11	0.2069	7.555	0.0784	0.1324
12	0.3883	4.2818	0.1582	0.2335
13	0.3824	4.0111	0.2081	0.2493
14	0.2452	6.0648	0.084	0.1649
15	0.1041	9.8785	0.0579	0.1012
16	0.287	4.5412	0.1419	0.2202
17	0.1192	6.804	0.0559	0.147
18	0.0774	12.6026	0.0522	0.0793
19	0.2392	5.6367	0.1153	0.1774
20	0.15	9.3954	0.0425	0.1064
21	0.2602	5.1856	0.154	0.1928

Table S3. Comparison of miRNAs levels and clinicopathological data as: age at time of diagnosis. sex and smoking history as well as histopathological characteristics of tumors (according to pTNM and AJCC classifications and NSCLC subtypes); Data are is presented as mean ± standard deviation (median).

Features	miR-23a	miR-let7i	miR-361	miR-1228
**Sex**				
F	0.047 ± 0.064 (0.024)	0.208 ± 0.175 (0.128)	0.179 ± 0.117 (0.178)	9.474 ± 8.028 (5.610)
M	0.059 ± 0.070 (0.032)	0.184 ± 0.197 (0.134)	0.202 ± 0.138 (0.171)	6.764 ± 3.955 (5.868)
**Age**				
≤65	0.075 ± 0.074 (0.070)	0.225 ± 0.241 (0.145)	0.240 ± 0.150 (0.187)	5.260 ± 2.13 (5.363)
>65	0.040 ± 0.057 (0.004)	0.170 ± 0.140 (0.125)	0160 ± 0.105 (0.159)	9.508 ± 6.873 (6.292)
**Histopathological type**				
SSC	0.057 ± 0.061 (0.039)	0.149 ± 0.121 (0.128)	0.199 ± 0.133 (0.186)	6.626 ± 3.88 (5.375)
ADC	0.055 ± 0.075 (0.010)	0.243 ± 0.235 (0.146)	0.192 ± 0.134 (0.177)	8.81 ± 7.306 (5.661)
**Tumor size; T**				
T1a + T1b + T1c	0.059 ± 0.076 (0.020)	0.206 ± 0.171 (0.170)	0.202 ± 0.101 (0.171)	5.949 ± 2.432 (5.842)
T2a + T2b	0.053 ± 0.073 (0.014)	0.170 ± 0.214 (0.115)	0.152 ± 0.077 (0.168)	9.348 ± 6.345 (5.974)
T3 + T4	0.058 ± 0.050 (0.068)	0.227 ± 0.190 (0.151)	0.189 ± 0.132 (0.170)	8.284 ± 7.504 (5.936)
**Intrathoracic lymph node involvement; N**				
N0	0.048 ± 0.070 (0.006)	0.178 ± 0.200 (0.112)	0.165 ± 0.088 (0.1445)	7.891 ± 4.591 (6.938)
N1	0.092 ± 0.055 (0.095)	0.218 ± 0.172 (0.162)	0.235 ± 0.110 (0.196)	4.871 ± 1.604 (5.098)
N2	0.002 ± 0.002 (0.001)	0.202 ± 0.201 (0.125)	0.228 ± 0.285 (0.114)	13.403 ± 11.723 (12.912)
**Malignant stage**				
Stage I	0.037 ± 0.065 (0.002)	0.122 ± 0.103 (0.084)	0.172 ± 0.098 (0.150)	7.722 ± 4.633 (6.721)
Stage II	0.096 ± 0.072 (0.090)	0.318 ± 0.254 (0.182)	0.190 ± 0.080 (0.186)	6.633 ± 4.378 (5.375)
Stage III	0.041 ± 0.051 (0.004)	0.176 ± 0.157 (0.146)	0.176 ± 0.132 (0.178)	9.510 ± 8.116 (5.610)
**Smoking history**				
current smokers	0.072 ± 0.076 (0.050)	0.236 ± 0.242 (0.137)	0.222 ± 0.176 (0.176)	7.495 ± 5.782 (5.719)
ex-smokers	0.038 ± 0.049 (0.007)	0.146 ± 0.121 (0.115)	0.169 ± 0.096 (0.173)	8.645 ± 6.686 (5.831)
never smokers	0.057 ± 0.085 (0.002)	0.220 ± 0.198 (0.181)	0.194 ± 0.080 (0.177)	5.705 ± 1.742 (5.661)
≤40 PYs	0.052 ± 0.043 (0.049)	0.158 ± 0.142 (0.137)	0.173 ± 0.107 (0.147)	8.348 ± 5.901 (6.864)
>40 PYs	0.067 ± 0.080 (0.039)	0.224 ± 0.236 (0.108)	0.223 ± 0.167 (0.187)	7.348 ± 6.435 (5.363)
